# Experimental Evaluation of GNSS Receiver Vulnerability to Spoofing and Jamming Using SDR-Based Testbed

**DOI:** 10.3390/s26144551

**Published:** 2026-07-17

**Authors:** Jan Dułowicz, Paweł Skokowski, Jan M. Kelner

**Affiliations:** 1 Cyber Defense Forces Component Command, 05-119 Legionowo, Poland; 2 Institute of Communications Systems, Faculty of Electronics, Military University of Technology, 00-908 Warsaw, Poland; pawel.skokowski@wat.edu.pl (P.S.); jan.kelner@wat.edu.pl (J.M.K.)

**Keywords:** GNSS, GPS, Galileo, jamming, spoofing, time-to-first-fix (TTFF), Skydel GNSS Simulation Software

## Abstract

**Highlights:**

**What are the main findings?**

- A reproducible software-defined radio (SDR)-based methodology was developed to experimentally compare the jamming and spoofing resilience of five commercial global navigation satellite system (GNSS) receivers under identical laboratory conditions.
- Navigation-fix continuity was shown to be an unreliable indicator of navigation integrity, while multi-band and multi-constellation receivers significantly delayed—but did not prevent—spoof acceptance.

**What are the implications of the main findings?**

- The proposed methodology provides a practical framework for repeatable benchmarking of GNSS receiver resilience against spoofing and jamming attacks.
- The results demonstrate the need for navigation-integrity assessment methods that extend beyond navigation-fix continuity, particularly in safety-critical and unmanned applications.

**Abstract:**

Global navigation satellite systems (GNSSs) are essential for navigation in aviation, transportation, and autonomous systems, yet they remain vulnerable to intentional interference such as jamming and spoofing. Unlike prior studies that primarily focus on positioning error, this work emphasizes acquisition-phase behavior, analyzing the impact of interference on time-to-first-fix (TTFF) and post-attack reacquisition time. A controlled and repeatable laboratory testbed based on software-defined radio (SDR) was developed to emulate Global Positioning System (GPS) L1 and Galileo E1 signals under multiple interference scenarios, including narrowband jamming, static spoofing, and dynamic spoofing. Five commercial GNSS receivers were evaluated under identical conditions. The results show that jamming causes an immediate loss of positioning capability, reducing the empirical navigation-fix probability to near zero and significantly increasing reacquisition time, with recovery-phase empirical fix probabilities ranging from 0.062 to 0.991 depending on receiver class. In contrast, spoofing maintains high attack-phase empirical navigation-fix probabilities ranging from 0.730 to 0.907 while introducing persistent and undetected errors. Static position spoofing was found to produce position offsets that persisted into the recovery phase, delaying the return to the authentic navigation solution. For most receivers, however, correct positioning was restored within the observation window. Multi-constellation spoofing further increases attack effectiveness, raising fix continuity by more than 0.15 compared to single-constellation cases. Multi-band receivers demonstrate increased resilience by delaying spoof acceptance by more than 4 min in extended scenarios, rather than preventing it entirely. The proposed methodology enables reproducible evaluation of GNSS receiver robustness and demonstrates that navigation-fix continuity alone is not a reliable indicator of navigation integrity during spoofing attacks. Overall, the results demonstrate that navigation-fix continuity alone cannot be regarded as a reliable indicator of navigation integrity and highlight the importance of complementary integrity-monitoring mechanisms for GNSS-dependent systems. The reported observations were obtained under controlled laboratory conditions and should be interpreted within the context of the adopted experimental methodology rather than as a direct representation of operational performance in real-world environments.

## 1. Introduction

Global navigation satellite systems (GNSSs) have become a critical infrastructure component for modern transportation, aviation, autonomous vehicles, unmanned aerial systems (UASs), and time-synchronization services. Their widespread adoption in civilian and military applications has simultaneously increased exposure to intentional radio-frequency interference, particularly jamming and spoofing attacks. Jamming raises the receiver noise floor and suppresses signal acquisition or tracking, whereas spoofing introduces counterfeit but plausible satellite signals that manipulate the receiver’s position, velocity, and time (PVT) solution while maintaining apparently valid operation [1,2,3,4,5]. Recent surveys indicate that spoofing has become one of the most significant threats to civil GNSS users because it can manipulate the position, velocity, and time solution while remaining considerably more difficult to detect than conventional jamming [6]. The disruptive effects of GNSS jamming and the integrity risks associated with spoofing have been widely recognized in both civilian and military applications [7,8]. In operational environments, such attacks may lead not only to navigation failure but also to subtle integrity degradation that remains undetected by the user or autonomous control system.

Previous research on GNSS interference has primarily focused on positioning error, signal authentication, spoofing detection methods, and navigation integrity degradation [2,4,5,9,10]. In particular, receiver autonomous integrity monitoring (RAIM)-based integrity monitoring and anti-spoofing techniques have been proposed to improve receiver resilience against counterfeit GNSS signals [2]. However, comparatively less attention has been devoted to acquisition-phase behavior and receiver recovery dynamics under contested radio-frequency (RF) conditions. Metrics such as time-to-first-fix (TTFF), fix continuity, spoof acceptance time, and post-attack re-acquisition are particularly important in applications characterized by frequent initialization cycles, intermittent satellite visibility, or strict operational readiness constraints, including unmanned aerial vehicles (UAVs), autonomous systems, and tactical platforms [2,3,9,11,12,13]. This is particularly relevant for UASs, where GNSS interference may be combined with communication-link jamming and other electronic-warfare techniques to disrupt autonomous operation [14]. Such metrics are particularly important for UAVs, whose navigation and communication systems increasingly rely on spectrum-constrained wireless environments and GNSS-based positioning [15,16]. In such systems, the ability of a receiver to rapidly recover from interference or resist false-lock conditions may be as important as positioning accuracy itself.

Recent studies increasingly employ software-defined radio (SDR) platforms for controlled GNSS threat emulation because they enable reproducible generation of jamming and spoofing scenarios under laboratory conditions [1,11,13,17,18,19,20,21]. The flexibility of SDR technology has also enabled the development of software-based GNSS receivers and signal-analysis platforms for navigation research and wireless-system experimentation [22,23]. The practicality of SDR-based spoofing has been demonstrated in several experimental studies. Horton and Ranganathan successfully developed a low-cost GPS spoofing platform capable of manipulating the navigation solution of a Da-Jiang Innovations (DJI) Matrice 100 UAV using commercially available SDR hardware and open-source software [24]. SDR-based approaches provide flexibility in generating synthetic GNSS constellations, manipulating satellite trajectories, and controlling interference timing and power levels while maintaining repeatability unavailable in open-air testing. Nevertheless, many existing experimental studies focus primarily on the effectiveness of attacks themselves rather than on comparative receiver behavior during the acquisition, attack, and recovery phases under identical controlled conditions.

The authors’ previous research has addressed several aspects of SDR-based radio-frequency interference relevant to GNSS resilience. Earlier work introduced an experimental methodology for evaluating the robustness of multi-band GNSS receivers to jamming [12], reviewed intentional interference techniques targeting GNSS signals and UAV communication links [25], and experimentally compared the effectiveness of selected GNSS jamming waveforms [26]. Complementary studies have also demonstrated the growing accessibility of SDR-enabled UAV jamming systems [18,19], practical GPS spoofing of UAVs [24], low-cost SDR-based spoofing implementations [27], and broader SDR-based GNSS spoofing instrumentation frameworks [1]. Collectively, these studies highlight both the increasing practicality of SDR-based navigation attacks and the need for reproducible methodologies for evaluating GNSS receiver resilience.

This paper presents an experimental SDR-based methodology for evaluating GNSS receiver vulnerability to spoofing and jamming under controlled laboratory conditions. Unlike prior studies focused mainly on navigation error, the proposed approach emphasizes acquisition-phase and recovery-phase behavior, including TTFF, fix continuity, spoof acceptance time, and post-attack re-acquisition dynamics. A repeatable wired RF testbed based on Safran’s Skydel GNSS Simulation Software (version 24.6.0) signal synthesis and a Universal Software Radio Peripheral (USRP) X310 SDR platform was developed to emulate GPS L1 and Galileo E1 signals under multiple interference scenarios, including narrowband jamming, dynamic altitude spoofing, and static position spoofing. Five commercial GNSS receivers representing different architectural classes were experimentally evaluated under identical conditions.

The main contributions of this work can be summarized as follows:

1. Development of a reproducible SDR-based GNSS interference evaluation platform supporting controlled spoofing and jamming experiments;
2. Comparative experimental analysis of acquisition, attack, and recovery behavior across multiple commercial GNSS receiver architectures;
3. Quantitative evaluation of TTFF, spoof acceptance time, fix continuity, and post-attack recovery metrics under single- and multi-constellation attacks;
4. Experimental demonstration that spoofing may preserve navigation-fix continuity while simultaneously introducing temporary false-position states that persist during the recovery phase after the attack has ended;
5. Experimental assessment of the resistance provided by multi-band receiver architectures against sustained spoofing attacks.

**Figure 1:**
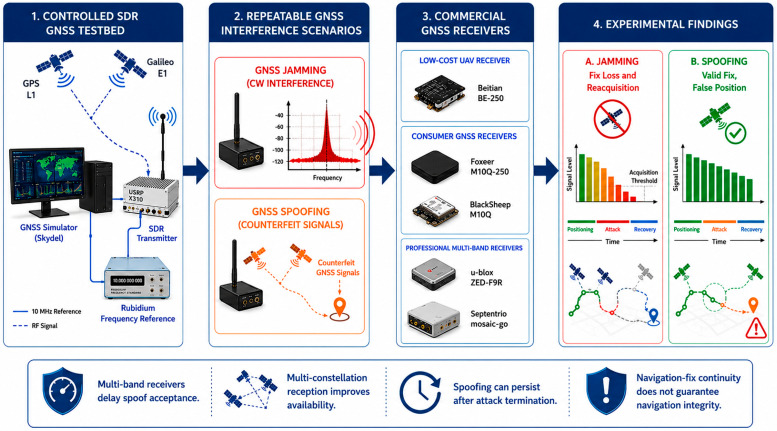
Graphical overview of the proposed SDR-based GNSS interference evaluation framework. The developed testbed enables controlled generation of GPS L1 and Galileo E1 spoofing and jamming scenarios using software-defined radio technology. Five commercial GNSS receivers representing different performance classes were evaluated. The experiments demonstrate that jamming primarily causes navigation-fix loss, whereas spoofing may maintain apparently valid navigation solutions while introducing falsified positioning information. Multi-band receivers exhibit improved resistance and delayed spoof acceptance but remain vulnerable during prolonged attacks.

Figure 1 provides a graphical overview of the developed SDR-based GNSS interference testbed, the investigated spoofing and jamming scenarios, the evaluated receiver categories, and the main experimental findings obtained in this study.

The remainder of the paper is organized as follows. Section 2 describes the experimental SDR-based GNSS testbed, receiver configurations, and measurement methodology. Section 3 presents the experimental results obtained for the jamming and spoofing scenarios, including acquisition, spoof acceptance, and recovery behavior analysis. Section 4 discusses the implications of the observed receiver responses and compares the resilience of the tested architectures. Finally, Section 5 concludes the paper and outlines directions for future work.

## 2. Materials and Methods

### 2.1. SDR-Based GNSS Testbed

**Figure 2:**
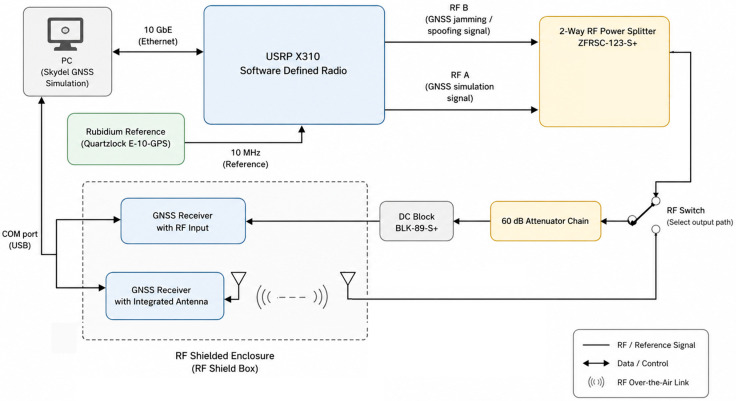
Conceptual diagram of the SDR-based GNSS testbed.

The experimental platform was developed to evaluate the vulnerability of commercial GNSS receivers to spoofing and jamming attacks under controlled and fully repeatable laboratory conditions. The testbed combines real-time GNSS signal synthesis, SDR transmission, and wired RF signal injection to ensure isolation from external radio-frequency interference and environmental variability. The overall architecture of the SDR-based GNSS interference platform is presented in Figure 2.

The GNSS signal generation subsystem was implemented using Safran’s Skydel GNSS Simulation Software running on a workstation equipped with an NVIDIA RTX 4050 graphics processing unit (GPU) and 64 GB random access memory (RAM) (Santa Clara, CA, USA). Real-time in-phase and quadrature (IQ) samples corresponding to selected GNSS constellations were generated using real ephemeris data for a predefined location and time. The synthesized IQ stream was transmitted using a USRP X310 SDR equipped with two UBX-160 daughterboards (Ettus Research, Austin, TX, USA).

The RF signal was injected into the tested receivers through a wired SubMiniature version A (SMA)-connected path consisting of an RF coupler, a direct current (DC) block, and a 60 dB attenuation chain. SDR clock stability was ensured using an external Quartzlock E-10-GPS rubidium 10 MHz frequency reference (HCD Research Ltd., Burgess Hill, WSX, UK).

Two experimental configurations were employed depending on the receiver architecture. The u-blox ZED-F9R (u-blox AG, Thalwil, Switzerland) and Septentrio mosaic-go receivers (Septentrio NV, Leuven, Belgium), which provide external RF antenna inputs, were evaluated using a conducted RF configuration incorporating an RF splitter, a 60 dB attenuation chain, and a DC block. In this configuration, the attenuation chain reduced the SDR output to a signal level representative of authentic GNSS reception at the receiver input while preventing excessive input power.

**Figure 3:**
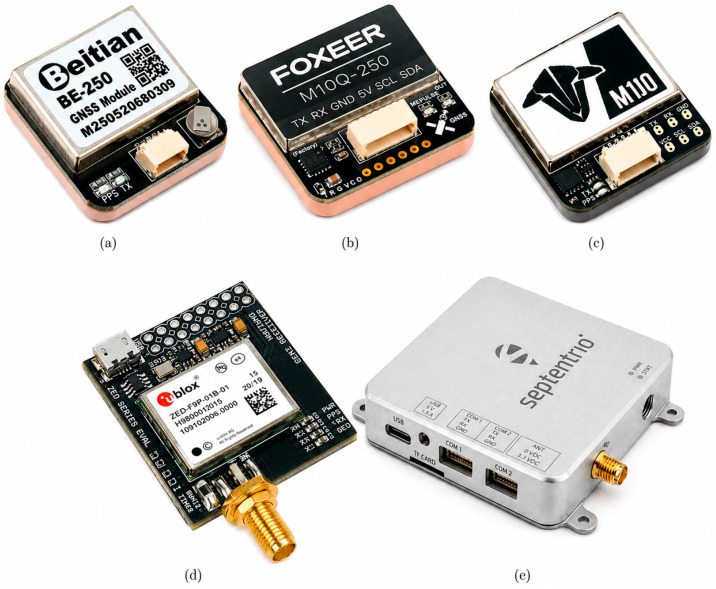
Three tested GNSS receivers with integrated antenna: (**a**) Beitian BE-250, (**b**) Foxeer M10Q-250, (**c**) BlackSheep M10Q, and two—with RF input: (**d**) u-blox ZED-F9R and (**e**) Septentrio mosaic-go.

The remaining three receivers (i.e., Beitian BE-250 (Shenzhen Beitian Communication Technology Co., Ltd., Shenzhen, China), Foxeer M10Q-250 (Foxeer Technology Co., Ltd., Shenzhen, China), and BlackSheep M10Q (Blacksheep Avionics Co., Ltd., Hong Kong, China)), equipped with integrated GNSS antennas, were evaluated using a radiated configuration inside the RF-shielded enclosure, as illustrated in Figure 2 and Figure 3. The SDR output power was experimentally adjusted and subsequently verified using a spectrum analyzer for each laboratory configuration to ensure reliable reception of the spoofing and jamming signals. In the radiated configuration, the SDR output was connected directly to the transmitting antenna located inside the RF-shielded enclosure. Owing to the short propagation distance and the absence of authentic satellite signals, the transmitted power was sufficient to ensure reliable reception by the receivers equipped with integrated GNSS antennas, and no RF power amplifier was required.

The RF output levels used during the experiments were verified using a spectrum analyzer prior to each measurement series. For the conducted configuration, the authentic GNSS signal level at the receiver input was approximately −90 dBm, whereas both the jamming and spoofing signals were adjusted to approximately −40 dBm. For the radiated configuration inside the RF-shielded enclosure, the corresponding signal levels were approximately −60 dBm for the authentic GNSS signal and −10 dBm for both the jamming and spoofing signals. These levels ensured reliable and repeatable receiver operation under the adopted laboratory conditions while preserving approximately the same interference-to-signal power ratio in both configurations.

The wired architecture ensured stable and reproducible signal conditions while eliminating uncontrolled external GNSS reception and ambient RF interference. The main hardware and RF configuration parameters are summarized in Table 1.

### 2.2. Tested GNSS Receivers

Receiver telemetry was collected through serial communication port (COM) interfaces and included satellite count, geographic coordinates, altitude, Coordinated Universal Time (UTC), and navigation-fix status. Five commercial GNSS receivers representing different architectural classes and market segments were evaluated in the experiments:

- Beitian BE-250;
- Foxeer M10Q-250;
- BlackSheep M10Q;
- u-blox ZED-F9R;
- Septentrio mosaic-go.

The tested receivers are presented in Figure 3, while their main characteristics are summarized in Table 2.

All receivers operated using factory-default firmware and configuration settings. Before each experimental run, the receivers were reset to enforce cold-start acquisition conditions. TTFF was defined as the elapsed time between receiver initialization and the first valid navigation fix.

### 2.3. Trajectory Generation and Signal Configuration

Dynamic receiver motion was simulated using trajectory files in comma-separated value (CSV) format generated with a custom software tool (position_generator.py). Each trajectory consisted of timestamped latitude, longitude, and altitude coordinates describing linear motion at constant velocity equal to 25 m/s (90 km/h). The simulation parameters included total trajectory duration, waypoint density, initial position, and altitude profile.

Two GNSS signal configurations were investigated:

- GPS L1 only (eight satellites);
- Combined GPS L1 (eight satellites) + Galileo E1 (eight satellites).

Both GPS L1 and Galileo E1 occupy the same RF center frequency of 1575.42 MHz. Consequently, a narrowband CW jammer centered at this frequency simultaneously affects both constellations.

Only open-service (OS) civilian GNSS signals were considered in this study. The spoofing scenarios were generated using Safran’s Skydel GNSS Simulation Software, which synthesizes GPS L1 C/A and Galileo E1 OS signals using publicly available signal specifications, navigation messages, and ephemeris data corresponding to the selected simulation epoch. Since all evaluated receivers operate exclusively on open-service civilian signals, encrypted military signals (e.g., GPS P(Y) or M-code) were neither required nor utilized. Consequently, the time-dependent characteristics of the navigation messages and spreading codes were inherently maintained by the simulator throughout all experiments, ensuring fully consistent and repeatable spoofing scenarios.

For the spoofing scenarios, the generated counterfeit GNSS signals were synchronized with the legitimate trajectory before attack activation and subsequently modified according to the selected spoofing profile. In altitude-spoofing scenarios, the spoofed signal gradually altered the reported altitude while preserving the legitimate horizontal trajectory. In static-position spoofing scenarios, the spoofed signal forced the receiver toward a fixed false geographic location.

**Figure 4:**
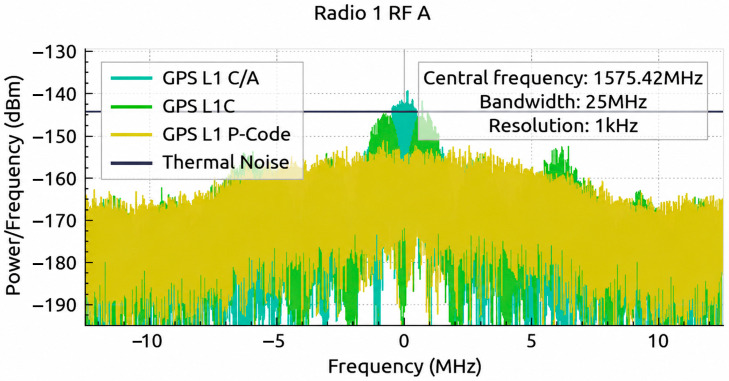
Amplitude–frequency characteristic of the legitimate GPS L1 signal.

**Figure 5:**
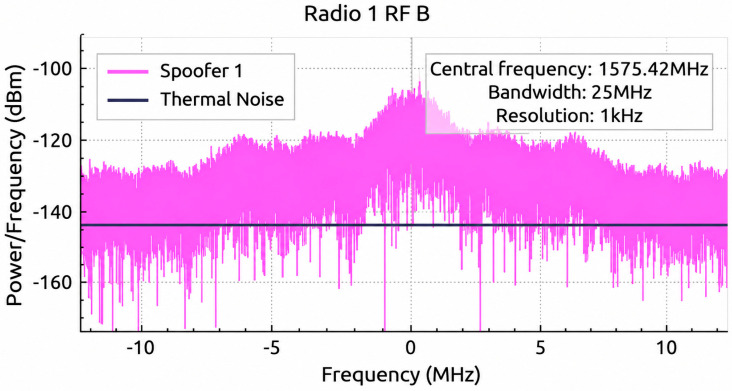
Amplitude–frequency characteristic of the spoofed GPS L1 signal.

**Figure 6:**
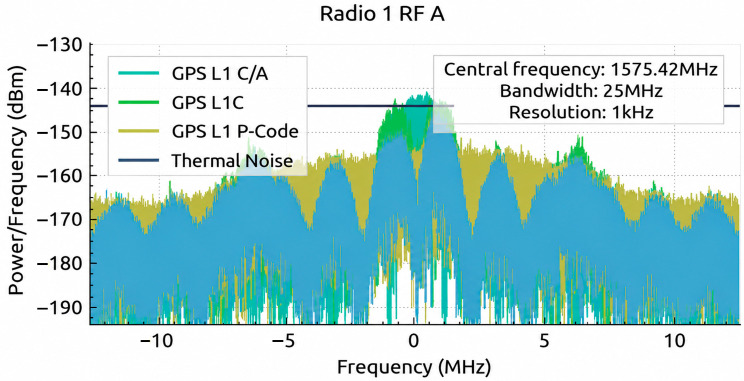
Amplitude–frequency characteristics of the legitimate GPS L1 + Galileo E1 signals.

**Figure 7:**
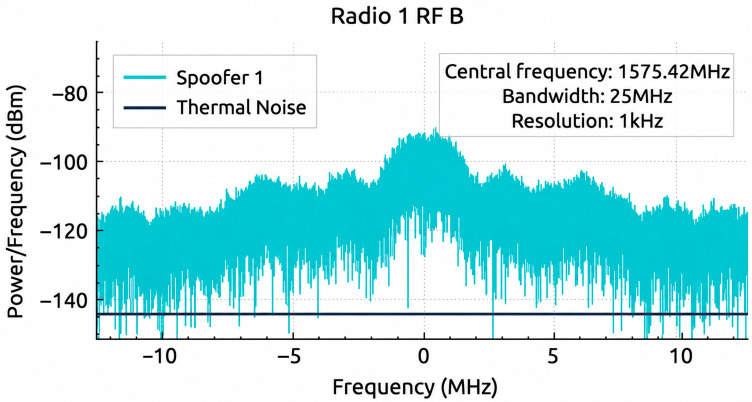
Amplitude–frequency characteristics of the spoofed GPS L1 + Galileo E1 signals.

**Figure 8:**
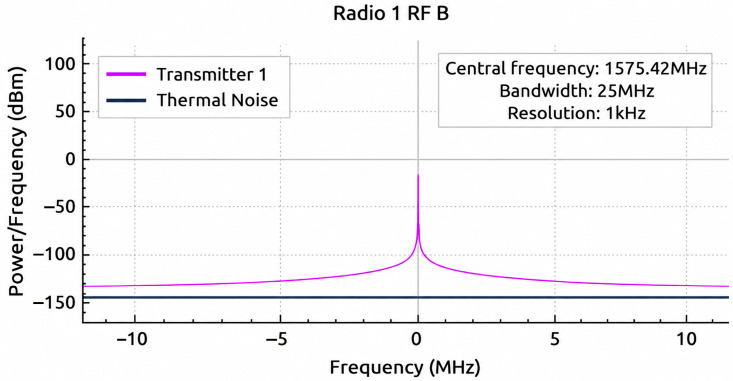
Spectrum of the narrowband CW jammer.

The amplitude–frequency characteristics of the legitimate and spoofed GNSS signals are presented in Figure 4, Figure 5, Figure 6 and Figure 7. Figure 8 presents the spectrum of the CW jammer used during the interference experiments.

### 2.4. Measurement Scenarios

Six interference scenarios were designed to evaluate GNSS receiver behavior under controlled spoofing and jamming conditions. The scenarios differed in signal configuration, interference type, and spoofing strategy while maintaining identical motion dynamics and RF infrastructure.

Each experiment consisted of three consecutive phases:

- Positioning phase;
- Attack phase;
- Recovery phase.

The positioning phase allowed the receiver to acquire a valid navigation fix under nominal GNSS conditions. During the attack phase, either spoofing or jamming interference was activated. Finally, the recovery phase evaluated the receiver’s ability to restore correct navigation performance after the interference source was removed.

The investigated measurement scenarios are summarized in Table 3.

In the jamming scenarios (1a–1b), the receivers moved along a linear trajectory at constant altitude equal to 100 m. The positioning phase covered the first 120 s of simulated flight, followed by a 60 s attack interval during which the CW jammer was activated. The remaining 120 s constituted the recovery phase.

In the spoofing scenarios (2a–2b and 3a–3b), the positioning phase covered the first 120 s of the experiment, followed by a 90 s spoofing interval and a 90 s recovery phase. In altitude-spoofing scenarios, the counterfeit signal gradually altered the receiver altitude from 100 m to approximately 350 m while maintaining the legitimate horizontal trajectory. In static-position spoofing scenarios, the counterfeit signal forced the receiver toward a fixed false geographic position while maintaining constant altitude.

To investigate delayed spoof acceptance and long-term recovery dynamics, selected spoofing scenarios were additionally extended to 15-min observations for the Beitian BE-250 and Septentrio mosaic-go receivers. The extended scenarios are summarized in Table 4.

In the extended experiments, the positioning, attack, and recovery phases each lasted 300 s, resulting in a total scenario duration of 900 s.

### 2.5. Evaluation Metrics

For each scenario, ten independent measurement repetitions were performed to evaluate acquisition stability, spoof acceptance behavior, navigation-fix availability, and post-attack recovery performance. For the 5-min scenarios, the time-dependent receiver responses presented in Section 3.1, Section 3.2 and Section 3.3 are shown as mean values calculated from ten independent measurement repetitions. The semi-transparent shaded regions represent the corresponding standard deviation at each time instant, thereby providing a graphical indication of measurement dispersion and repeatability.

The primary evaluation metrics are summarized in Table 5.

TTFF represents the elapsed time between receiver initialization and acquisition of the first valid navigation solution:

(1) $$TTFF=t_{fix}-t_{start},$$

where $t_{start}$ denotes receiver initialization time and $t_{fix}$ is the time of the first valid navigation fix.

Spoof acceptance time represents the elapsed time between activation of the spoofing signal and the first occurrence of a falsified navigation solution:

(2) $$T_{spoof}=t_{false\_fix}-t_{spoof\_start},$$

where $t_{spoof\_start}$ and $t_{false\_fix}$ are the spoof activation time and first detected spoofed navigation solution, respectively.

For altitude-spoofing scenarios, spoof acceptance corresponded to persistent deviation toward the counterfeit altitude profile. For static-position spoofing scenarios, spoof acceptance was defined as persistent convergence toward the spoofed geographic coordinates.

Recovery time represents the elapsed time between removal of the interference signal and restoration of the legitimate navigation solution:

(3) $$T_{recovery}=t_{correct\_fix}-t_{attack\_end},$$

where $t_{attack\_end}$ means the time at which the interference signal is removed and $t_{correct\_fix}$ the time at which the receiver restores the authentic navigation solution.

If recovery was not observed within the measurement window, the recovery time was treated as undefined for the corresponding repetition.

The empirical fix probability, $P_{fix}\left(t\right)$, represents the probability of maintaining a valid navigation fix at time instant t across repeated measurement runs:

(4) $$P_{fix}\left(t\right)=N_{fix}\left(t\right)/N_{runs},$$

where $N_{runs}$ and $N_{fix}\left(t\right)$ are the number of measurement runs in which the receiver reported a valid navigation fix at time t and the total number of repeated measurements, respectively.

For the experiments presented in this work $N_{runs}=10$,

(5) $$0\leq P_{fix}\left(t\right)\leq 1,$$

where $P_{fix}\left(t\right)=0$ indicates complete loss of navigation fix (i.e., $N_{fix}\left(t\right)=0$) and $P_{fix}\left(t\right)=1$ indicates uninterrupted fix availability across all repetitions (i.e., $N_{fix}\left(t\right)=N_{runs}$).

## 3. Results

The experimental results are organized according to the measurement scenarios defined in Section 2.4. Scenarios 1a and 1b evaluate receiver behavior under narrowband jamming. Scenarios 2a and 2b analyze altitude spoofing under GPS L1-only and combined GPS L1 + Galileo E1 configurations. Scenarios 3a and 3b evaluate static-position spoofing. Finally, the extended 15-min experiments investigate long-term spoofing dynamics for the Beitian BE-250 and Septentrio mosaic-go receivers under selected altitude and static-position spoofing scenarios.

The evaluated receivers and the corresponding experimental scenarios are summarized in Table 6.

### 3.1. Jamming Scenarios

The first group of experiments evaluated GNSS receiver behavior under narrowband continuous-wave interference centered at 1575.42 MHz. Scenario 1a used the GPS L1 signal configuration, whereas Scenario 1b used the combined GPS L1 + Galileo E1 configuration. The analysis focused on time to first fix, loss of navigation-fix availability during the attack phase, and post-attack reacquisition behavior.

**Figure 9:**
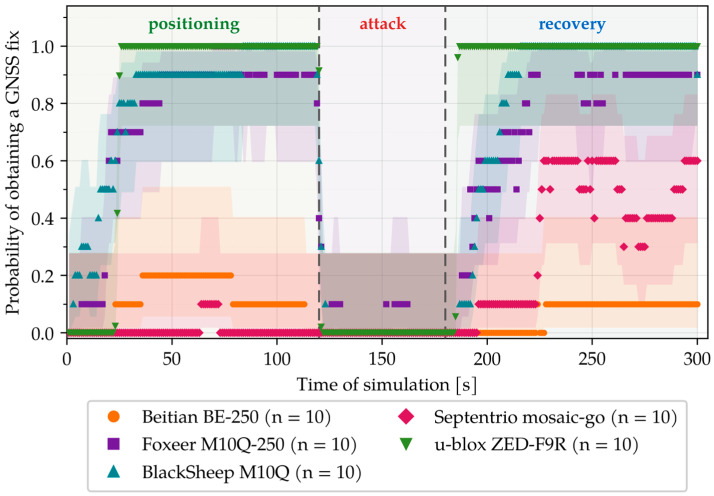
Empirical fix probability versus simulation time for Scenario 1a: GPS L1 jamming. Markers indicate mean values from ten repetitions, and shaded regions represent the corresponding standard deviation.

**Figure 10:**
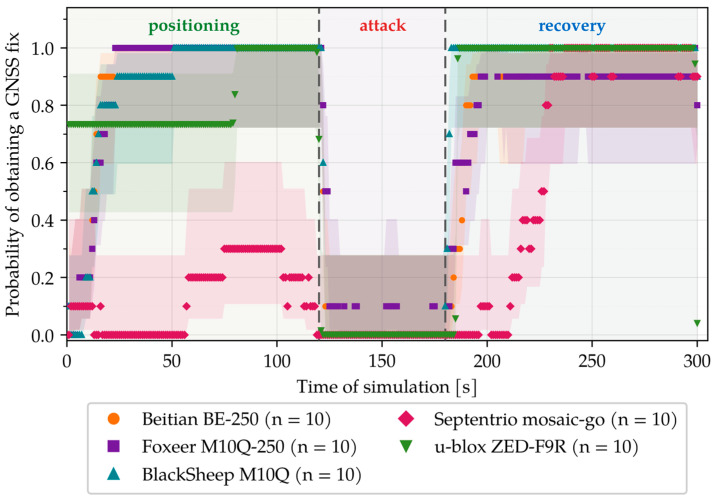
Empirical fix probability versus simulation time for Scenario 1b: GPS L1 + Galileo E1 jamming. Markers indicate mean values from ten repetitions, and shaded regions represent the corresponding standard deviation.

Figure 9 and Figure 10 illustrate the empirical fix probabilities obtained for Scenarios 1a (GPS L1 reception was disrupted by the CW jammer) and 1b (the receiver was exposed to jamming under the combined GPS L1 + Galileo E1 configuration), respectively.

The results for Scenario 1a show that jamming causes an almost immediate degradation in navigation-fix availability after the attack is activated. As shown in Figure 9, the empirical fix probability rapidly decreases immediately after the jammer is enabled (120 s), reaching values close to zero for most receivers during the attack interval. This behavior reflects the receivers’ inability to maintain reliable signal tracking under the applied continuous-wave interference. Following jammer deactivation, the receivers gradually reacquire the authentic GNSS signals. However, the recovery rate differs considerably between receiver architectures. The low-cost receivers exhibit rapid fix loss, while the u-blox ZED-F9R demonstrates the strongest post-attack reacquisition behavior. The Septentrio mosaic-go shows slower acquisition and recovery in the 5-min observation window, which is consistent with its longer initialization behavior.

Compared with Scenario 1a, the additional Galileo E1 signals (Scenario 1b) improved navigation-fix continuity for several receivers, particularly during the initial stage of the attack (see Figure 10). The increased number of tracked satellites provided greater measurement redundancy and delayed complete loss of navigation fix. Nevertheless, the applied jammer affected the common L1/E1 frequency, and prolonged interference eventually reduced the fix probability for all tested receivers.

Overall, the jamming results confirm that narrowband interference primarily affects receiver availability rather than navigation integrity. In all investigated receiver architectures, the CW jammer caused rapid degradation of signal tracking and navigation-fix availability, while receiver recovery occurred only after the interference source had been removed. The observed differences among receivers were therefore associated mainly with reacquisition performance rather than with resistance to the applied interference itself. Unlike spoofing, jamming does not produce a plausible but false navigation solution; instead, it reduces or eliminates the receiver’s ability to maintain a valid fix.

### 3.2. Altitude Spoofing Scenarios

The second group of experiments evaluated altitude spoofing. Scenario 2a used GPS L1 only, whereas Scenario 2b used the combined GPS L1 + Galileo E1 configuration. In both cases, the spoofing signal was designed to alter the reported altitude while preserving the horizontal motion profile.

**Figure 11:**
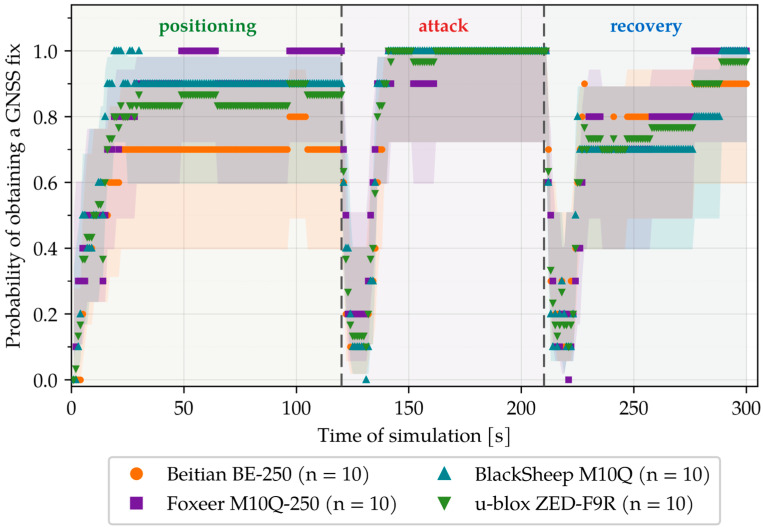
Empirical fix probability versus simulation time for Scenario 2a: GPS L1 altitude spoofing. Markers indicate mean values from ten repetitions, and shaded regions represent the corresponding standard deviation.

**Figure 12:**
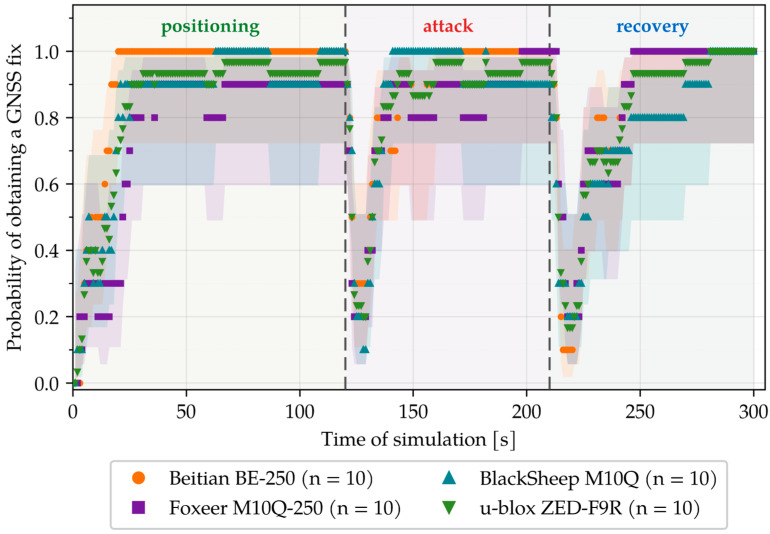
Empirical fix probability versus simulation time for Scenario 2b: GPS L1 + Galileo E1 altitude spoofing. Markers indicate mean values from ten repetitions, and shaded regions represent the corresponding standard deviation.

Figure 11 and Figure 12 present the empirical fix probabilities for Scenarios 2a (under GPS L1 altitude spoofing) and 2b (under combined GPS L1 + Galileo E1 altitude spoofing), respectively.

**Figure 13:**
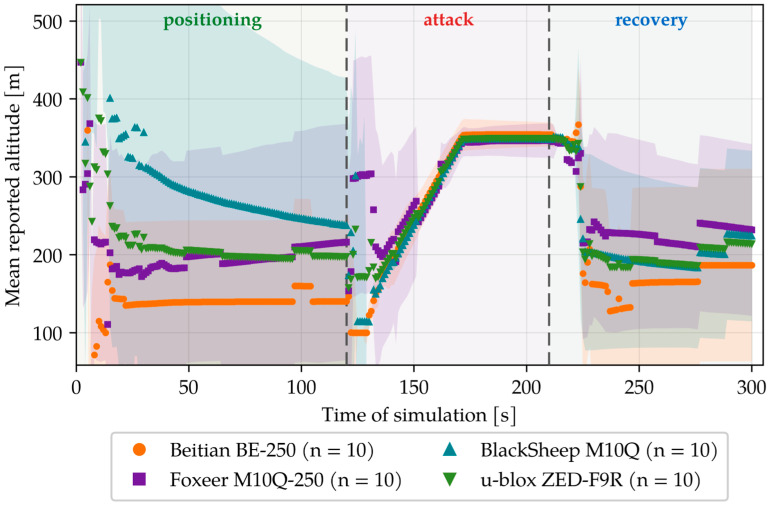
Mean reported altitude versus simulation time for Scenario 2a: GPS L1 altitude spoofing. Markers indicate mean values from ten repetitions, and shaded regions represent the corresponding standard deviation.

The results for Scenario 2a indicate that altitude spoofing does not cause the same fix-loss behavior as jamming. As illustrated in Figure 11, the empirical fix probability remains high throughout the spoofing interval for all tested receivers, indicating uninterrupted receiver operation. However, Figure 13 simultaneously shows a progressive deviation of the reported altitude toward the counterfeit trajectory. The quantitative metrics summarized in Table 7 confirm that spoof acceptance occurred within several tens of seconds for all receivers except the Septentrio mosaic-go. Consequently, receiver availability alone cannot be regarded as evidence of correct navigation.

**Figure 14:**
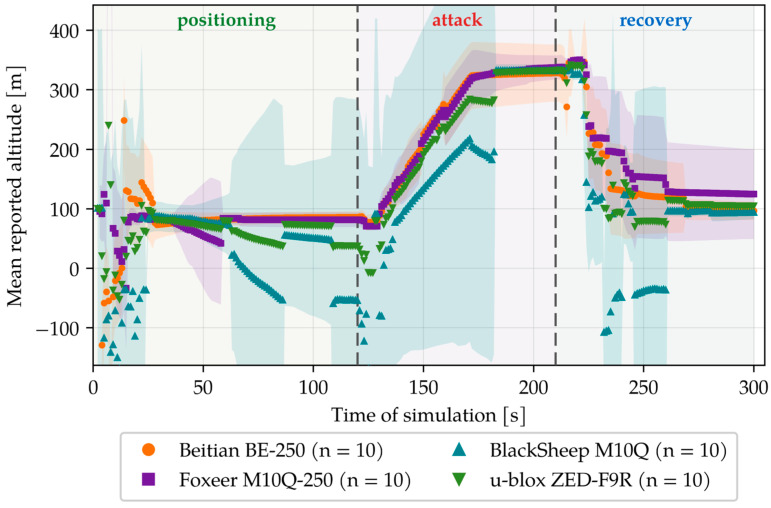
Mean reported altitude versus simulation time for Scenario 2b: GPS L1 + Galileo E1 altitude spoofing. Markers indicate mean values from ten repetitions, and shaded regions represent the corresponding standard deviation.

The combined GPS L1 + Galileo E1 spoofing configuration produced even higher navigation-fix continuity during the attack phase (see Figure 12). The altitude trajectories shown in Figure 14 demonstrate that the counterfeit trajectory was generally followed more consistently than in the GPS-only scenario. These observations indicate that multi-constellation spoofing provides a more stable counterfeit navigation environment, increasing the probability that receivers will accept the spoofed solution.

Figure 13 and Figure 14 illustrate the mean reported altitudes for Scenarios 2a and 2b, respectively.

In Scenario 2a, after spoof activation, the tested receivers gradually converge toward the falsified altitude profile. The transition is not equivalent to loss of fix; instead, the receivers maintain navigation solutions while reporting incorrect altitude values. After the spoofing signal is removed, the receivers begin returning toward the nominal altitude, although the recovery behavior differs across receiver types.

The combined GPS L1 + Galileo E1 spoofing case (i.e., Scenario 2b) produces a similarly effective altitude manipulation. Compared with Scenario 2a, the spoofed altitude trajectory is more consistently followed by the receivers, while recovery after attack termination may be slower. This confirms that adding Galileo E1 to the spoofed signal can increase the stability of the falsified navigation solution.

The main altitude-spoofing performance metrics are summarized in Table 7.

Table 7 highlights considerable differences in spoof resistance among the tested receiver architectures. While the single-band receivers accepted the spoofed solution within approximately 15–40 s, the professional multi-band Septentrio mosaic-go did not accept the counterfeit altitude within the 5-min observation window, demonstrating substantially higher resistance to this attack scenario.

The altitude-spoofing results demonstrate that spoofing attacks can preserve apparent receiver availability while corrupting the navigation solution. Therefore, monitoring only the presence of a valid fix is insufficient for spoofing detection.

### 3.3. Static Position Spoofing Scenarios

The third group of experiments evaluated static-position spoofing. Scenario 3a used GPS L1 only, whereas Scenario 3b used the combined GPS L1 + Galileo E1 configuration. In these scenarios, the spoofing signal forced the receiver toward a fixed false geographic position while the legitimate trajectory continued.

**Figure 15:**
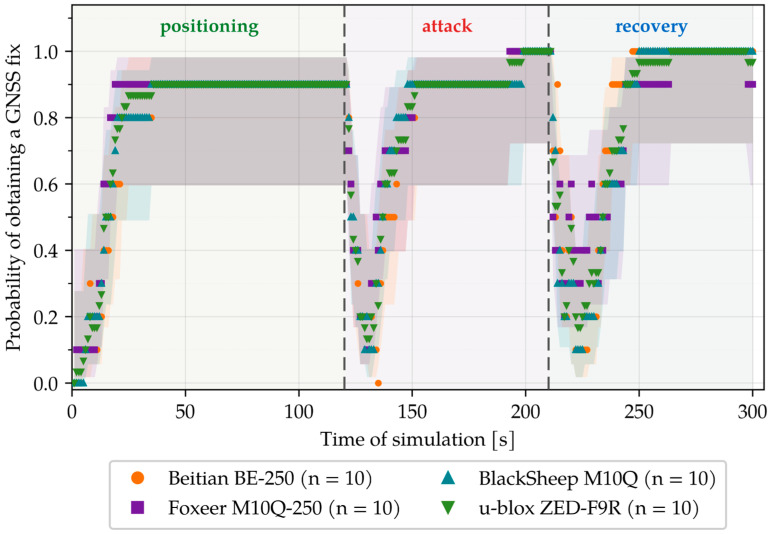
Empirical fix probability versus simulation time for Scenario 3a: GPS L1 static-position spoofing. Markers indicate mean values from ten repetitions, and shaded regions represent the corresponding standard deviation.

**Figure 16:**
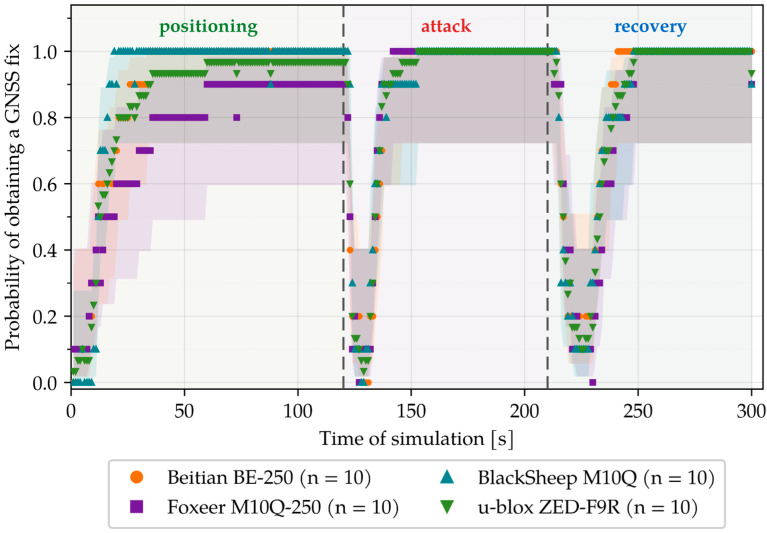
Empirical fix probability versus simulation time for Scenario 3b: GPS L1 + Galileo E1 static-position spoofing. Markers indicate mean values from ten repetitions, and shaded regions represent the corresponding standard deviation.

Figure 15 and Figure 16 depict the empirical fix probabilities for Scenarios 3a (under GPS L1 static-position spoofing) and 3b (under combined GPS L1 + Galileo E1 static-position spoofing), respectively.

**Figure 17:**
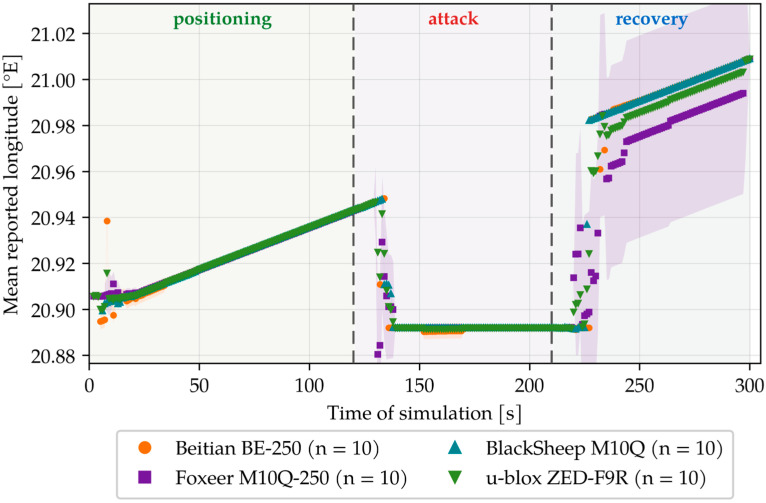
Mean reported longitude versus simulation time for Scenario 3a: GPS L1 static-position spoofing. Markers indicate mean values from ten repetitions, and shaded regions represent the corresponding standard deviation.

The results for Scenario 3a show that the receivers maintain a high probability of preserving a valid navigation fix throughout the spoofing interval. As shown in Figure 15, the empirical fix probability remained close to unity for most receivers during the attack phase despite the presence of counterfeit GNSS signals. At the same time, Figure 17 demonstrates that the reported longitude gradually converged toward the spoofed position instead of the legitimate trajectory. The quantitative metrics summarized in Table 8 further confirm that spoof acceptance occurred within several tens of seconds for all receivers except the Septentrio mosaic-go. These observations indicate that the spoofing attack was generally accepted without causing loss of navigation lock, clearly distinguishing spoofing from the jamming scenarios discussed in Section 3.1.

**Figure 18:**
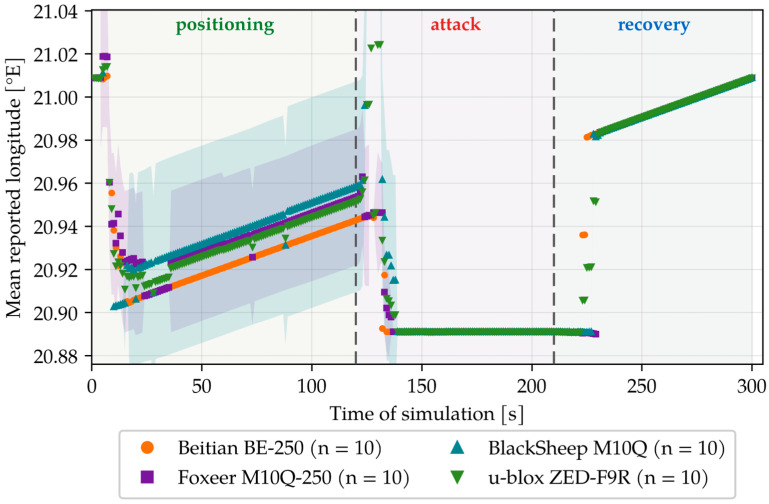
Mean reported longitude versus simulation time for Scenario 3b: GPS L1 + Galileo E1 static-position spoofing. Markers indicate mean values from ten repetitions, and shaded regions represent the corresponding standard deviation.

The combined GPS L1 + Galileo E1 spoofing scenario produced even greater navigation-fix continuity (see Figure 16). Moreover, the longitude trajectories shown in Figure 18 indicate that the receivers remained locked to the counterfeit position after spoof activation, with greater consistency than in the GPS-only configuration. These observations indicate that multi-constellation spoofing increases both the plausibility and stability of the falsified navigation solution.

Figure 17 and Figure 18 show the mean reported longitudes for Scenario 3a and 3b, respectively.

In Scenario 3a, during the positioning phase, the receivers follow the legitimate trajectory. After spoof activation, all tested receivers converge toward the injected static longitude. Importantly, the falsified position temporarily persists after the spoofing signal is removed. Although the receivers do not return immediately to the legitimate trajectory, most of them recover the correct longitude within the recovery phase, as reflected by the recovery times summarized in Table 8.

The combined GPS L1 + Galileo E1 static-position spoofing scenario confirms the persistence of the false-position lock. The receivers accept the spoofed position and remain close to the falsified longitude during the initial part of the recovery phase. This demonstrates that static-position spoofing may temporarily affect the receiver state even after the attack has ended. However, with the exception of the Septentrio mosaic-go receiver, all tested devices recovered the authentic navigation solution within the observation window.

The main performance metrics for static-position spoofing are summarized in Table 8.

As summarized in Table 8, all single-band receivers rapidly accepted the spoofed position and subsequently recovered after spoof removal, whereas the Septentrio mosaic-go exhibited substantially greater resistance, confirming the benefits of multi-band signal processing against spoofing attacks.

The static-position spoofing results show that a receiver may remain locked to a false position even after spoof transmission has ceased. This persistent false-position lock is particularly important for autonomous systems, where recovery cannot be assumed solely because the interference source has disappeared.

### 3.4. Extended Long-Duration Experiments

The 5-min experiments provide statistically repeatable fix-probability results based on repeated runs. However, they are too short to fully capture slow receiver dynamics, especially for professional multi-band receivers with longer initialization and validation procedures. Therefore, selected spoofing scenarios were additionally evaluated in 15-min experiments for two receivers: the low-cost Beitian BE-250 and the professional Septentrio mosaic-go. The extended scenarios included altitude spoofing and static-position spoofing under both GPS L1-only and combined GPS L1 + Galileo E1 configurations.

**Figure 19:**
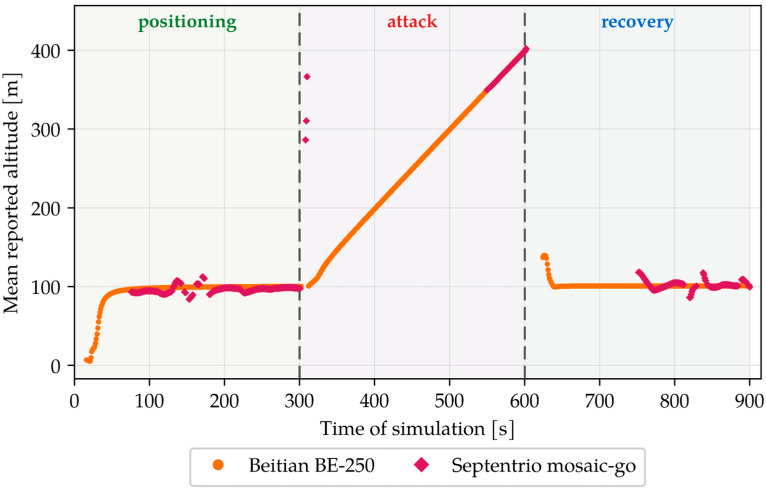
Mean reported altitude versus simulation time for Beitian BE-250 and Septentrio mosaic-go in extended Scenario 2a: GPS L1 altitude spoofing.

**Figure 20:**
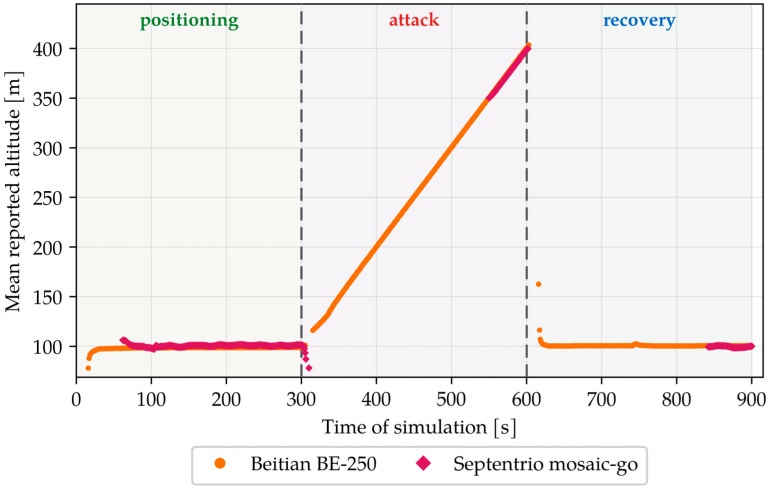
Mean reported altitude versus simulation time for Beitian BE-250 and Septentrio mosaic-go in extended Scenario 2b: GPS L1 + Galileo E1 altitude spoofing.

Figure 19 and Figure 20 present the extended altitude-spoofing results for Scenarios 2a (using GPS L1) and 2b (using the combined GPS L1 + Galileo E1 configuration), respectively.

In Scenario 2a, the Beitian BE-250 accepts the falsified altitude shortly after spoof activation and follows the spoofed altitude trajectory. The Septentrio mosaic-go also becomes affected by the spoofing signal, but its response is less continuous, indicating intermittent acceptance and rejection of the falsified solution. This behavior suggests that multi-band receiver processing can disturb spoof tracking even when it does not completely prevent altitude deception.

Scenario 2b highlights the advantage of the professional multi-band receiver. While the Beitian BE-250 accepts the spoofed altitude shortly after attack activation, the Septentrio mosaic-go delays spoof acceptance for most of the attack window. This demonstrates that multi-band receiver architecture does not guarantee immunity, but it can significantly increase the time required for a successful spoofing takeover.

**Figure 21:**
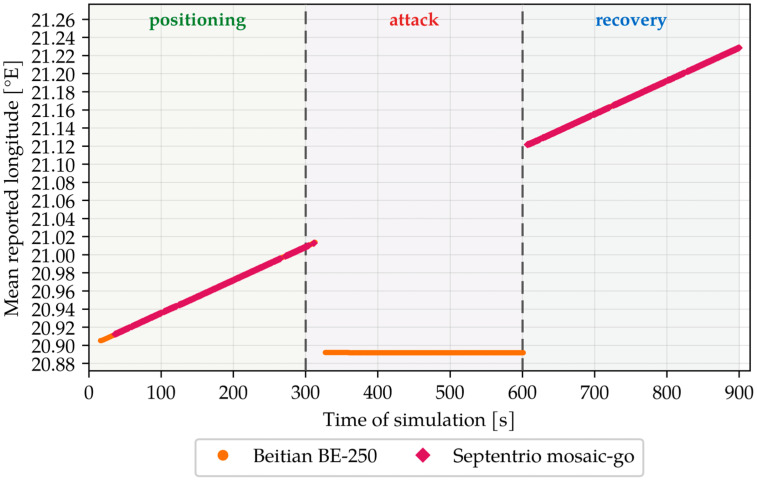
Mean reported longitude versus simulation time for Beitian BE-250 and Septentrio mosaic-go in extended Scenario 3a: GPS L1 static-position spoofing.

**Figure 22:**
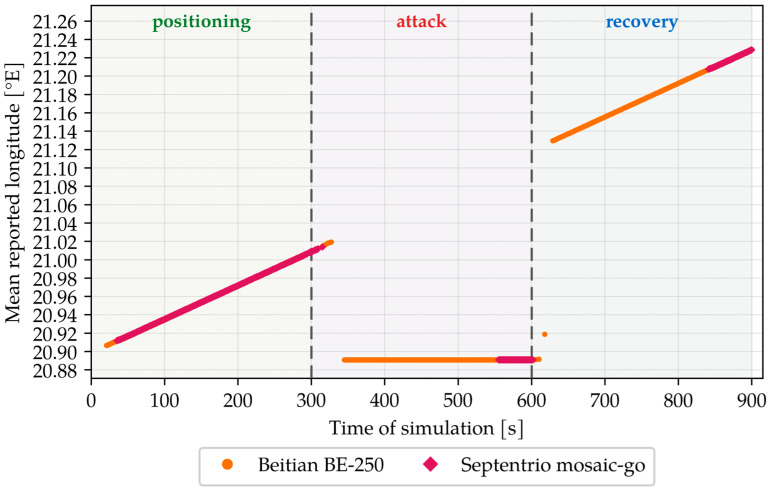
Mean reported longitude versus simulation time for Beitian BE-250 and Septentrio mosaic-go in extended Scenario 3b: GPS L1 + Galileo E1 static-position spoofing.

Figure 21 and Figure 22 depict the extended static-position spoofing results for Scenarios 3a (using GPS L1) and 3b (using the combined GPS L1 + Galileo E1 configuration), respectively.

In Scenario 3a, the Beitian BE-250 accepts the spoofed static position and remains locked to the falsified longitude throughout the recovery phase. In contrast, the Septentrio mosaic-go largely rejects the GPS L1-only static-position spoofing attempt and avoids reporting a falsified position during the attack interval. This indicates that the professional multi-band receiver can preserve position integrity by refusing to provide a fix rather than outputting a false navigation solution.

In Scenario 3b, the combined-constellation spoofing signal is more effective. The Beitian BE-250 again accepts the false static position and remains locked to it after attack termination. The Septentrio mosaic-go delays spoof acceptance substantially, but eventually also converges toward the falsified longitude. Once the false position is accepted, no recovery to the correct trajectory is observed within the 300-s recovery phase.

Unlike the 5-min experiments, the extended observations reveal receiver behaviors that cannot be captured within shorter measurement intervals. In particular, the delayed spoof acceptance exhibited by the Septentrio mosaic-go demonstrates that increased spoof resistance does not necessarily imply complete immunity. Instead, sophisticated spoofing signals may eventually be accepted after prolonged exposure.

The extended spoofing experiments are summarized in Table 9.

The extended experiments reveal receiver dynamics that are not visible in the 5-min repeated measurements. The Beitian BE-250 rapidly accepts both altitude and static-position spoofing and, in static-position scenarios, does not recover the correct longitude within the observation window. The Septentrio mosaic-go provides substantially stronger resistance, especially against GPS L1-only static-position spoofing, but it does not provide absolute immunity. In combined-constellation spoofing, even the professional multi-band receiver eventually accepts the falsified solution after prolonged exposure.

### 3.5. Comparative Analysis

The complete set of results demonstrates a fundamental difference between jamming and spoofing. Jamming primarily causes loss of navigation availability, whereas spoofing preserves apparently valid fixes while corrupting the navigation solution. This distinction is critical because a receiver may appear operational during spoofing even though its reported altitude or position is false.

The overall phase-level empirical fix probability $P_{fix}\left(t\right)$ across the 5-min scenarios is summarized in Table 10. The phase-level values reported in Table 7 were calculated as the temporal mean of $P_{fix}\left(t\right)$ over the corresponding positioning, attack, or recovery interval.

The results presented in Table 9 and Table 10 quantitatively confirm the trends observed in Figure 19, Figure 20, Figure 21 and Figure 22. The extended experiments clearly distinguish between receivers that immediately accepted spoofed signals and those capable of delaying spoof acceptance by several minutes.

The comparative results show that the addition of Galileo E1 generally improves acquisition and fix continuity, but this improvement has different implications depending on the type of interference. Under jamming, additional constellation availability can support reacquisition outside the attack interval. Under spoofing, however, a more complete counterfeit constellation may increase the stability and continuity of the false navigation solution.

Overall, the results demonstrate that multi-band and professional receiver architectures improve resilience mainly by delaying or rejecting spoof acceptance, not by guaranteeing complete immunity. The most important practical implication is that navigation-fix continuity alone cannot be used as a reliable integrity indicator. Robust GNSS-dependent systems require additional consistency checks, such as signal-quality monitoring, multi-sensor validation, time consistency checks, and explicit reinitialization procedures after suspected spoofing exposure.

The experimental observations presented above provide the basis for the broader discussion of receiver resilience, practical implications, comparison with previous studies, and limitations presented in the following section.

## 4. Discussion

The experimental results presented in Section 3 consistently demonstrate that navigation-fix continuity alone cannot be regarded as a reliable indicator of navigation integrity. The following discussion interprets these observations in the context of receiver architecture, compares them with previously published studies, and considers their practical implications and limitations. Whereas jamming primarily degrades signal reception and often results in an immediate loss of positioning capability, spoofing attempts to maintain normal receiver operation while gradually introducing erroneous navigation information. Consequently, the availability of a valid navigation fix cannot be considered a sufficient indicator of positioning integrity.

The conducted measurements revealed substantial differences among the evaluated receivers despite their common purpose and support for modern GNSS constellations. These differences were particularly evident during spoofing attacks, where receiver-specific signal-processing algorithms and integrity-monitoring mechanisms strongly influenced attack susceptibility and recovery behavior.

### 4.1. Receiver Architecture and Spoofing Susceptibility

The results presented in Section 3.2, Section 3.3 and Section 3.4 (i.e., for Scenarios 2 and 3) indicate that receiver architecture plays a significant role in determining resistance to GNSS spoofing.

The single-frequency receivers equipped with integrated antennas generally exhibited the highest susceptibility to spoofing attacks. In several experiments, these devices maintained continuous navigation fixes while accepting counterfeit navigation solutions, resulting in substantial position deviations without any apparent indication of abnormal operation.

By contrast, the multi-frequency and multi-constellation receivers demonstrated improved robustness. The u-blox ZED-F9R and Septentrio mosaic-go receivers frequently showed shorter spoofing acceptance intervals, reduced position deviations, and faster recovery after the termination of the attack signal. The observed behavior is likely associated with the use of additional frequency bands, enhanced signal-quality monitoring, and more sophisticated navigation-filtering algorithms.

Nevertheless, the results also demonstrate that advanced receiver architectures do not provide complete immunity against spoofing. Under sufficiently realistic attack conditions, all evaluated receivers exhibited some degree of navigation-solution corruption. Therefore, increased receiver complexity should be regarded as a factor improving resilience rather than eliminating vulnerability.

### 4.2. Comparison with Previous Studies

The observations obtained in the spoofing experiments presented in Figure 11, Figure 12, Figure 13, Figure 14, Figure 15, Figure 16, Figure 17, Figure 18, Figure 19, Figure 20, Figure 21 and Figure 22 and summarized in Table 7, Table 8, Table 9 and Table 10 are consistent with previous reports indicating that spoofing attacks often preserve navigation-fix continuity while simultaneously corrupting the navigation solution [9,12]. In contrast to jamming, which typically causes a rapid degradation of positioning performance, spoofing can remain unnoticed because the receiver continues reporting apparently valid navigation data.

These observations agree with the pioneering work of Humphreys et al. [28], who demonstrated that counterfeit GNSS signals can manipulate receiver position estimates while maintaining normal receiver operation. Similar conclusions were reported by Psiaki and Humphreys [4], who emphasized the unique challenge posed by spoofing attacks due to their ability to generate plausible yet incorrect navigation solutions.

The present findings are also consistent with comprehensive reviews of GNSS spoofing and anti-spoofing technologies, which highlight that spoofing attacks may preserve receiver availability while degrading navigation integrity [2]. Furthermore, the use of controlled SDR-based testbeds for evaluating receiver behavior under repeatable spoofing conditions has been recognized as an effective methodology for assessing spoofing susceptibility and mitigation techniques [1].

The extended experiments performed in this study additionally indicate that multi-frequency and multi-constellation receiver architectures may significantly delay spoof acceptance, although they do not guarantee complete protection against carefully generated spoofing signals. These observations support previous findings that enhanced receiver architectures improve spoofing resilience primarily by increasing resistance time rather than eliminating vulnerability altogether [2].

An important observation emerging from the conducted experiments is the large variability among commercial receivers exposed to identical interference conditions. Even when subjected to the same spoofing signal, receivers frequently exhibited different acceptance thresholds, recovery times, and navigation-solution deviations. This finding suggests that proprietary receiver implementations may have an impact comparable to hardware architecture itself.

Overall, the results reinforce previous findings regarding the challenges posed by GNSS spoofing while providing additional experimental evidence on the influence of receiver architecture, constellation diversity, and attack duration on spoofing resilience.

### 4.3. Practical Implications for GNSS Resilience Assessment

The experimental results presented in Section 3, particularly those obtained for the spoofing scenarios (see Figure 11, Figure 12, Figure 13, Figure 14, Figure 15, Figure 16, Figure 17, Figure 18, Figure 19, Figure 20, Figure 21 and Figure 22 and Table 7, Table 8, Table 9 and Table 10), highlight several practical considerations for evaluating GNSS receiver security. The following discussion summarizes the principal engineering implications of the observed receiver behavior, focusing on appropriate performance metrics, receiver architecture, and practical recommendations for assessing resilience to spoofing.

First, navigation-fix availability should not be used as the sole performance metric during spoofing assessments. Several receivers maintained valid fixes while producing significantly incorrect position estimates. Therefore, spoofing resilience should be evaluated using both availability metrics and navigation-integrity indicators.

Second, the results demonstrate that controlled SDR-based testbeds provide an effective means for evaluating receiver behavior under repeatable interference conditions. The proposed experimental platform enabled direct comparison of multiple receiver architectures under identical scenarios, thereby reducing variability associated with uncontrolled environmental factors.

Third, the extended experiments performed using the Beitian BE-250 and Septentrio mosaic-go receivers indicate that receiver diversity remains an important consideration when designing resilient GNSS-dependent systems. Devices employing different architectures may respond differently to the same interference source, suggesting that heterogeneous receiver deployments may provide additional operational robustness.

Overall, the presented methodology can support both receiver benchmarking and the development of future anti-spoofing and anti-jamming techniques.

### 4.4. Study Limitations

Several limitations of the present study should be acknowledged.

First, the experiments were performed using five representative commercial receivers and a limited set of controlled interference scenarios. Although the selected devices cover multiple receiver architectures and application domains, the obtained results should not be generalized to all commercially available GNSS receivers without further experimental validation.

Second, all measurements were conducted under controlled laboratory conditions using software-generated spoofing and jamming signals. Although this approach ensures excellent repeatability and enables direct comparison of receiver behavior under identical interference conditions, it does not fully reproduce the complexity of real operational environments. In particular, real-world interference events may involve dynamic receiver motion, multipath propagation, varying signal power levels, antenna effects, and the simultaneous presence of authentic satellite signals. Consequently, the quantitative results reported in this study should be interpreted within the context of the adopted laboratory methodology. Accordingly, the presented results should not be directly extrapolated to operational environments without additional validation under realistic propagation and interference conditions.

Third, the analysis focused primarily on navigation-fix probability, spoof acceptance behavior, and post-attack recovery performance. Additional receiver-level indicators, such as carrier-to-noise density ratio (C/N_0_), pseudo-range residuals, and integrity-monitoring statistics, were not available for all tested receivers and therefore remained outside the scope of this work.

## 5. Conclusions

This study experimentally evaluated the susceptibility of five commercial GNSS receivers to jamming and spoofing using a controlled SDR-based testbed capable of generating GPS L1 and Galileo E1 signals under repeatable laboratory conditions.

The results demonstrate that jamming and spoofing affect GNSS receivers in fundamentally different ways. While jamming primarily degrades signal availability and frequently results in loss of navigation fix, spoofing can maintain apparently valid receiver operation while simultaneously corrupting the navigation solution. Consequently, navigation-fix continuity alone cannot be regarded as a reliable indicator of navigation integrity.

The experiments further showed that multi-constellation spoofing is generally more effective than single-constellation spoofing. The simultaneous spoofing of GPS L1 and Galileo E1 increased fix continuity during the attack phase and promoted more stable acceptance of falsified navigation solutions.

The standard 5-min scenarios demonstrated the general response of all five receivers to jamming and spoofing, including fix loss during jamming, spoof acceptance behavior, and post-attack recovery dynamics. The extended 15-min scenarios, performed for the Beitian BE-250 and Septentrio mosaic-go receivers, further showed that increased receiver sophistication may delay spoof acceptance but does not necessarily guarantee immunity under prolonged exposure.

The extended 15-min experiments revealed substantial differences between receiver architectures. The professional multi-band Septentrio mosaic-go receiver demonstrated significantly greater resistance to spoofing than the single-band Beitian BE-250, particularly in terms of spoof acceptance time and rejection of certain spoofing scenarios. Nevertheless, even advanced receiver architectures did not provide complete immunity against prolonged and carefully generated spoofing signals.

A particularly important observation was the temporary persistence of false-position solutions during the recovery phase following spoofing termination. Although all evaluated receivers eventually recovered correct positioning within the observation window, several required a noticeable recovery interval before returning to the authentic navigation solution. During this recovery period, receivers remained locked to the spoofed navigation solution despite removal of the counterfeit signal, demonstrating that successful spoofing may continue to affect navigation performance for a limited time after the attack has ended.

Overall, the results confirm that receiver architecture, constellation diversity, and attack duration strongly influence spoofing resilience. Although the reported results were obtained under controlled laboratory conditions, the proposed SDR-based methodology provides a practical and repeatable framework for the comparative evaluation of GNSS receiver resilience. The developed methodology may also support future validation studies under more realistic operational conditions and contribute to the development and assessment of anti-spoofing techniques.

Future work will extend the experimental campaign to a larger set of GNSS receivers and more sophisticated interference scenarios, including dynamic trajectories, coordinated multi-constellation spoofing attacks, and advanced signal-authentication techniques. Particular attention will also be devoted to the development of quantitative spoofing-detection metrics that can be applied across heterogeneous receiver architectures.

## Figures and Tables

**Table 1:** Experimental platform configuration.

Parameter	Value
GNSS simulator	Safran’s Skydel GNSS Simulation Software
SDR platform	USRP X310
SDR daughterboards	2 × UBX-160
Reference clock	Quartzlock E-10-GPS
GNSS signals	GPS L1, Galileo E1
RF center frequency (MHz)	1575.42
RF attenuation (dB)	60
RF connection	Wired SMA
Jammer type	Narrowband continuous-wave (CW)
Number of repetitions	10

**Table 2:** Main characteristics of the evaluated GNSS receivers.

Receiver	Architecture	Frequency Support	Market Segment
Beitian BE-250	Single-band	GPS L1	Low-cost
Foxeer M10Q-250	Single-band	GPS L1	UAV/navigation
BlackSheep M10Q	Single-band	GPS L1	UAV/navigation
u-blox ZED-F9R	Multi-band	L1/L2	High-end navigation
Septentrio mosaic-go	Multi-band	Multi-frequency	Professional

**Table 3:** Overview of the experimental interference scenarios.

Scenario	Signal Configuration	Attack Type	Attack Duration (s)	Total Duration (s)
1a	GPS L1	CW jamming	60	300
1b	GPS L1 + Galileo E1	CW jamming	60	300
2a	GPS L1	Altitude spoofing	90	300
2b	GPS L1 + Galileo E1	Altitude spoofing	90	300
3a	GPS L1	Static position spoofing	90	300
3b	GPS L1 + Galileo E1	Static position spoofing	90	300

**Table 4:** Extended 15-min measurement scenarios.

Scenario	Tested Receivers	Purpose
2a	Beitian BE-250, Septentrio mosaic-go	Long-term altitude spoofing response
2b	Beitian BE-250, Septentrio mosaic-go	Multi-constellation altitude spoofing resistance
3a	Beitian BE-250, Septentrio mosaic-go	Persistent false-position analysis
3b	Beitian BE-250, Septentrio mosaic-go	Multi-band spoof resistance evaluation

**Table 5:** Definitions of the evaluation metrics used in this study.

Metric Symbol	Metric Name	Definition
TTFF	TTFF	Time between receiver initialization and first valid fix
$T_{spoof}$	Spoof acceptance time	Time between spoof activation and false-solution lock
$T_{recovery}$	Recovery time	Time required to recover correct navigation after attack removal
$P_{fix}\left(t\right)$	Empirical fix probability	Empirical probability of valid navigation-fix availability

**Table 6:** Overview of evaluated receiver architectures and scenarios.

Receiver	Jamming	Altitude Spoofing	Static Spoofing	Extended Tests
Beitian BE-250	✓	✓	✓	✓
Foxeer M10Q-250	✓	✓	✓	✗
BlackSheep M10Q	✓	✓	✓	✗
u-blox ZED-F9R	✓	✓	✓	✗
Septentrio mosaic-go	✓	✓	✓	✓

**Table 7:** Altitude spoofing performance metrics for Scenarios 2a and 2b.

Receiver	Scenario	TTFF (s)	Spoof Acceptance (s)	Persistent Spoof After Attack	Recovery Time (s)
Beitian BE-250	2a	17	17	No	27
Beitian BE-250	2b	14	24	No	25
Foxeer M10Q-250	2a	16	15	No	31
Foxeer M10Q-250	2b	15	19	No	32
BlackSheep M10Q	2a	12	14	No	23
BlackSheep M10Q	2b	19	18	No	24
u-blox ZED-F9R	2a	18	31	No	28
u-blox ZED-F9R	2b	16	38	No	30
Septentrio mosaic-go	2a	>120	>120	Yes	N/A
Septentrio mosaic-go	2b	>120	>120	Yes	N/A

**Table 8:** Static-position spoofing performance metrics for Scenarios 3a and 3b.

Receiver	Scenario	TTFF (s)	Spoof Acceptance (s)	Persistent Spoof After Attack	Recovery Time (s)
Beitian BE-250	3a	17	16	No	18
Beitian BE-250	3b	12	12	No	13
Foxeer M10Q-250	3a	14	15	No	11
Foxeer M10Q-250	3b	12	13	No	21
BlackSheep M10Q	3a	15	17	No	16
BlackSheep M10Q	3b	12	19	No	18
u-blox ZED-F9R	3a	15	17	No	17
u-blox ZED-F9R	3b	12	20	No	15
Septentrio mosaic-go	3a	>120	>120	Yes	N/A
Septentrio mosaic-go	3b	>120	>120	Yes	N/A

**Table 9:** Extended 15-min spoofing scenario comparison for Beitian BE-250 and Septentrio mosaic-go.

Receiver	Scenario	Spoof Acceptance (s)	Recovery Time (s)	Main Observation
Beitian BE-250	2a	25	24	Rapid altitude spoof acceptance
Septentrio mosaic-go	2a	250	45	Intermittent spoof acceptance
Beitian BE-250	2b	23	19	Rapid altitude spoof acceptance
Septentrio mosaic-go	2b	250	242	Strongly delayed spoof acceptance
Beitian BE-250	3a	27	21	Persistent false-position lock
Septentrio mosaic-go	3a	Not accepted	N/A	Spoof rejected/no false position reported
Beitian BE-250	3b	25	29	Persistent false-position lock
Septentrio mosaic-go	3b	255	241	Delayed acceptance followed by persistent false-position lock

**Table 10:** Empirical fix probability across 5-min scenarios and measurement phases.

Scenario	Phase	Beitian BE-250	Foxeer M10Q-250	BlackSheep M10Q	U-Blox ZED-F9R	Septentrio Mosaic-Go
1a	Positioning	0.112	0.750	0.806	0.803	0.008
	Attack	0.000	0.025	0.007	0.000	0.000
	Recovery	0.062	0.766	0.838	0.958	0.345
1b	Positioning	0.899	0.887	0.865	0.820	0.131
	Attack	0.030	0.072	0.028	0.000	0.000
	Recovery	0.926	0.864	0.991	0.950	0.644
2a	Positioning	0.651	0.854	0.843	0.783	—
	Attack	0.813	0.832	0.827	0.824	—
	Recovery	0.732	0.734	0.689	0.719	—
2b	Positioning	0.916	0.761	0.840	0.839	—
	Attack	0.903	0.837	0.884	0.875	—
	Recovery	0.839	0.844	0.749	0.811	—
3a	Positioning	0.785	0.807	0.785	0.792	—
	Attack	0.730	0.762	0.751	0.748	—
	Recovery	0.770	0.802	0.791	0.788	—
3b	Positioning	0.810	0.784	0.776	0.790	—
	Attack	0.896	0.907	0.903	0.902	—
	Recovery	0.866	0.877	0.873	0.872	—

The dash (“—”) in Scenarios 2a–3b indicates that the multi-band Septentrio mosaic-go failed to acquire a fix during the 120-s positioning phase in any of the ten runs—a consequence of its longer cold-start initialization procedure—making phase-resolved fix probability undefined for these scenarios. This limitation is addressed separately through the 15-min extended observations.

## Data Availability

The original contributions presented in this study are included in the article. Further inquiries can be directed to the corresponding author.

## Abbreviations

The following abbreviations are used in this manuscript:

C/N_0_	carrier-to-noise density ratio
COM	communication port
CSV	comma-separated values
CW	continuous-wave
DC	direct current
DJI	Da-Jiang Innovations
GNSS	global navigation satellite system
GPS	Global Positioning System
GPU	graphics processing unit
IQ	in-phase and quadrature
OS	open service
PVT	position, velocity, and time
RAIM	receiver autonomous integrity monitoring
RAM	random access memory
RF	radio-frequency
SDR	software-defined radio
SMA	SubMiniature version A
TTFF	time-to-first-fix
UAS	unmanned aerial system
UAV	unmanned aerial vehicle
USRP	Universal Software Radio Peripheral
UTC	Coordinated Universal Time

## References

[1] Schmidt E., Ruble Z., Akopian D., Pack D.J. (2019). Software-Defined Radio GNSS Instrumentation for Spoofing Mitigation: A Review and a Case Study. IEEE Trans. Instrum. Meas..

[2] Wu Z., Zhang Y., Yang Y., Liang C., Liu R. (2020). Spoofing and Anti-Spoofing Technologies of Global Navigation Satellite System: A Survey. IEEE Access.

[3] Kerns A.J., Shepard D.P., Bhatti J.A., Humphreys T.E. (2014). Unmanned Aircraft Capture and Control via GPS Spoofing. J. Field Robot..

[4] Psiaki M.L., Humphreys T.E. (2016). GNSS Spoofing and Detection. Proc. IEEE.

[5] Jafarnia-Jahromi A., Broumandan A., Nielsen J., Lachapelle G. (2012). GPS Vulnerability to Spoofing Threats and a Review of Antispoofing Techniques. Int. J. Navig. Obs..

[6] Meng L., Yang L., Yang W., Zhang L. (2022). A Survey of GNSS Spoofing and Anti-Spoofing Technology. Remote Sens..

[7] Han S., Luo D., Meng W., Li C. (2016). Antispoofing RAIM for Dual-Recursion Particle Filter of GNSS Calculation. IEEE Trans. Aerosp. Electron. Syst..

[8] Zhang C., Shui L., Huang H., Chen Q., Liu H., Jiao H., Feng L. (2016). Research on the Mechanism and the Impact of Jamming on Beidou Software Receiver. Proceedings of the 2016 IEEE Chinese Guidance, Navigation and Control Conference (CGNCC).

[9] Zidan J., Adegoke E.I., Kampert E., Birrell S.A., Ford C.R., Higgins M.D. (2021). GNSS Vulnerabilities and Existing Solutions: A Review of the Literature. IEEE Access.

[10] Wesson K., Rothlisberger M., Humphreys T. (2012). Practical Cryptographic Civil GPS Signal Authentication. NAVIGATION.

[11] Park S., Kim H.T., Lee S., Joo H., Kim H. (2021). Survey on Anti-Drone Systems: Components, Designs, and Challenges. IEEE Access.

[12] Zmysłowski D., Kryk M., Kelner J.M. (2023). Testing GNSS Receiver Robustness for Jamming. Aviat. Secur. Issues.

[13] Mohsan S.A.H., Othman N.Q.H., Li Y., Alsharif M.H., Khan M.A. (2023). Unmanned Aerial Vehicles (UAVs): Practical Aspects, Applications, Open Challenges, Security Issues, and Future Trends. Intell. Serv. Robot..

[14] Brito A., Sebastião P., Souto N. (2019). Jamming for Unauthorized UAV Operations-Communications Link. Proceedings of the 2019 International Young Engineers Forum (YEF-ECE).

[15] Jasim M.A., Shakhatreh H., Siasi N., Sawalmeh A.H., Aldalbahi A., Al-Fuqaha A. (2022). A Survey on Spectrum Management for Unmanned Aerial Vehicles (UAVs). IEEE Access.

[16] Lv H., Liu F., Yuan N. (2021). Drone Presence Detection by the Drone’s RF Communication. J. Phys. Conf. Ser..

[17] Chamola V., Kotesh P., Agarwal A., Naren, Gupta N., Guizani M. (2021). A Comprehensive Review of Unmanned Aerial Vehicle Attacks and Neutralization Techniques. Ad Hoc Netw..

[18] Šimon O., Götthans T. (2022). A Survey on the Use of Deep Learning Techniques for UAV Jamming and Deception. Electronics.

[19] Šimon O., Götthans T., Popela M. (2022). Commercial UAV Jamming Possibilities. Proceedings of the 2022 32nd International Conference Radioelektronika (RADIOELEKTRONIKA).

[20] Pärlin K., Alam M.M., Le Moullec Y. (2018). Jamming of UAV Remote Control Systems Using Software Defined Radio. Proceedings of the 2018 International Conference on Military Communications and Information Systems (ICMCIS).

[21] Di Pietro R., Oligeri G., Tedeschi P. (2019). JAM-ME: Exploiting Jamming to Accomplish Drone Mission. Proceedings of the 2019 IEEE Conference on Communications and Network Security (CNS).

[22] Juang J.-C., Tsai C.-T., Chen Y.-H. (2013). Development of a PC-Based Software Receiver for the Reception of Beidou Navigation Satellite Signals. J. Navig..

[23] Haluza M., Čechák J. (2016). Analysis and Decoding of Radio Signals for Remote Control of Drones. Proceedings of the 2016 New Trends in Signal Processing (NTSP).

[24] Horton E., Ranganathan P. (2018). Development of a GPS Spoofing Apparatus to Attack a DJI Matrice 100 Quadcopter. J. Glob. Position. Syst..

[25] Dułowicz J., Skokowski P., Kelner J.M. (2025). Survey on Intentional Interference Techniques of GNSS Signals and Radio Links between Unmanned Aerial Vehicle and Ground Control Station. TransNav Int. J. Mar. Navig. Saf. Sea Transp..

[26] Dułowicz J., Skokowski P., Kelner J.M. (2025). Energy-Efficient Jammers for GNSS Signals. Proceedings of the 2025 Signal Processing Symposium (SPSympo).

[27] Margana B.S., Achanta D.S., Songala K.K., Ammana S.R. (2021). A Simple SDR Based Method to Spoof Low-End GPS Aided Drones for Securing Locations. Proceedings of the 2021 IEEE International Conference on Robotics, Automation, Artificial-Intelligence and Internet-of-Things (RAAICON).

[28] Humphreys T.E., Ledvina B.M., Psiaki M.L., O’Hanlon B.W., Kintner P.M. (2008). Assessing the Spoofing Threat: Development of a Portable GPS Civilian Spoofer. Proceedings of the 2008 21st International Technical Meeting of the Satellite Division of The Institute of Navigation (ION GNSS).

